# Characteristics Analysis of F_1_ Hybrids between Genetically Modified *Brassica napus* and *B*. *rapa*

**DOI:** 10.1371/journal.pone.0162103

**Published:** 2016-09-15

**Authors:** Soo-In Sohn, Young-Ju Oh, Kyeong-Ryeol Lee, Ho-Cheol Ko, Hyun-Suk Cho, Yeon-Hee Lee, Ancheol Chang

**Affiliations:** 1 Department of Agricultural Biotechnology, National Institute of Agricultural Sciences, 370 Nongsaengmyeong-ro, Wansan-gu, Jeonju, North Jeolla Province, 54874, Republic of Korea; 2 Institute for Future Environmental Ecology Co., Ltd, 5, Palbok 1-gil, Deokjin-gu, Jeonju, North Jeolla Province, 54883, Republic of Korea; 3 National Agrobiodiversity Center, National Institute of Agricultural Sciences, 370 Nongsaengmyeong-ro, Wansan-gu, Jeonju, North Jeolla Province, 54874, Republic of Korea; United States Department of Agriculture, UNITED STATES

## Abstract

A number of studies have been conducted on hybridization between transgenic *Brassica napus* and *B*. *rapa* or backcross of F_1_ hybrid to their parents. However, trait changes must be analyzed to evaluate hybrid sustainability in nature. In the present study, *B*. *rapa* and transgenic (*BrAGL20*) *B*. *napus* were hybridized to verify the early flowering phenomenon of F_1_ hybrids, and F_1_ hybrid traits were analyzed to predict their impact on sustainability. Flowering of F_1_ hybrid has been induced slightly later than that of the transgenic *B*. *napus*, but flowering was available in the greenhouse without low temperature treatment to young plant, similar to the transgenic *B*. *napus*. It is because the *BrAGL20* gene has been transferred from transgenic *B*. *napus* to F_1_ hybrid. The size of F_1_ hybrid seeds was intermediate between those of *B*. *rapa* and transgenic *B*. *napus*, and ~40% of F_1_ pollen exhibited abnormal size and morphology. The form of the F_1_ stomata was also intermediate between that of *B*. *rapa* and transgenic *B*. *napus*, and the number of stomata was close to the parental mean. Among various fatty acids, the content of erucic acid exhibited the greatest change, owing to the polymorphism of parental *FATTY ACID ELONGASE 1* alleles. Furthermore, F_2_ hybrids could not be obtained. However, BC_1_ progeny were obtained by hand pollination of *B*. *rapa* with F_1_ hybrid pollen, with an outcrossing rate of 50%.

## Introduction

The concern associated with the cultivation of genetically modified (GM) crops is the increase of weedy or invasive crops as the transgenes are transferred to related species [1,2]. The environmental risk of GM crops can be evaluated, based on the concept of substantial equivalence, by comparing GM and non-GM crops in a natural-like environment, and if any significant differences are observed, the likelihood of negative impact can be used as a measure of risk [3]. Also, the Cartagena Protocol on Biosafety III suggests that assessing the environmental risk of genetically modified organisms (GMOs) should be conducted by identifying the organisms’ new genotypic and phenotypic characteristics that could negatively impact the biodiversity of its potential habitat. In addition, although gene flow and introgression are natural processes, gene flow from transgenic plants to wild relatives complicates the potential introgression of new traits.

To date, researchers have attempted to predict the consequences of hybridization and introgression between transgenic crops and related species [4–8]. Halfhill et al. [7] reported that crop-weed hybrids have lower fitness and competitive ability than their parents, regardless of transgene introgression, and such decreases in hybrid fitness are suggested to result from the introduction of crop genes, rather than from the introduction of transgenes. Therefore, the fitness of hybrids derived from transgenic crops in natural ecosystems should be estimated with the sustainability of the transgenic hybrids in the ecosystem through the analysis of transgene introgression course and result, as well as trait changes in the hybrids, owing to the genetic load of crop-derived genes.

Considering the fact that Korea has imported transgenic *B*. *napus* seeds and has exported *B*. *rapa* seeds, it is very important to assess the gene flow of transgenic *B*. *napus* to *B*. *rapa* and to determine the ecological impacts of hybridization on the unintentional release of transgenes, in order to establish appropriate biosafety measures. *B*. *rapa* is one of the ancestral species of *B*. *napus*. A number of studies have shown that the two species can hybridize [9–12], that spontaneous F_1_ hybrids occur in nature [13], and that backcrossing occurs when the hybrids are grown in the vicinity of *B*. *rapa* [13–19]. Although the average fitness of the F_1_ hybrid and first backcross (BC_1_) progeny is generally low [9], *B*. *rapa*-like plants that contain transgenes can be found even after single backcrosses [10,18–23]. In addition, there have been more studies of hybrids between *B*. *rapa* and transgenic *B*. *napus* than hybrids between other *Brassicaceae* and transgenic *B*. *napus*; however, due to the genetic diversity of *B*. *rapa* the sustainability and ecological effects of crop genes introduced *via* pollen movement are poorly understood.

The regulation of flowering time is an important trait in terms of crop productivity. *AGAMOUS-LIKE* 20 (*AGL20*) is known to encode a MADS-box transcription factor that integrates signals from multiple flowering pathways, including those related to photoperiod, temperature, hormones, and age [24]. A study in *Arabidopsis* demonstrated that *AGL20* expression plays an important role in determining flowering time and affects many floral induction pathways [25]. When *AGL20* from *B*. *rapa* was introduced into *B*. *napus*, the flowering time was advanced by up to 105 d, compared to non-transgenic *B*. *napus* [26], and although the early flowering transgenic *B*. *napus* has not yet been commercialized, it may be useful for assessing environmental sustainability, with regard to transgene expression and gene flow between transgenic *B*. *napus* and *B*. *rapa*.

This study aimed to investigate the expression pattern of target gene in the F_1_ hybrid between early flowering transgenic *B*. *napus* and *B*. *rapa*, analyze the environmental sustainabillity through analyzing morphological characteristics and fertility of F_1_ hybrid, and anticipate the unintended effects to the environment by the analyses of fatty acids of F_1_ seed. To do this, expression of target gene and other flowering-related genes was analyzed in F_1_ hybrids and parents. We also have compared the morphological characteristics of seeds, pollens and stomatal apparatus between F_1_ hybrids and parents. Fertility of F_1_ hybrids was investigated *via* self-pollination and backcrossing with *B*. *rapa*. Also, the content of erucic acid and nucleotide sequences of genes related to the synthesis of erucic acid were analyzed in the F_1_ hybrid seeds.

## Materials and Methods

### Plant materials

Early flowering transgenic *Brassica napus* L. ‘Youngsan’ (AACC, 2n = 38) was transformed with CAMV 35S-regulated *bar* and *BrAGL20* [26], and *B*. *napus* L. ‘Youngsan’ and *B*. *rapa* L. ssp. *pekinensis* ‘Jangkang’ (AA, 2n = 20) seeds were obtained from the National Agrobiodiversity Center (Jeonju, Republic of Korea).

### Outcrossing rate of F_1_ hybrid between *B*. *rapa* and transgenic *B*. *napus*

Cross experiments were conducted in the GMO greenhouse of NAAS (National Academy of Agricultural Science) located in Suwon, Korea. Interspecific crossability was determined using transgenic *B*. *napus* as the pollen donor and *B*. *rapa* as the seed parent, by means of artificial emasculation and crossing. For each cross combination, 100~200 flowers from 15 separate plants were crossed. Self-pollinated *B*. *napus* and transgenic *B*. *napus* were used as controls, and the pod number, number of seeds per pod, and seed weight were investigated after harvest. To obtain the F_2_ seeds 20 F_1_ plants were self-pollinated. In addition, BC_1_ progeny were produced using a F_1_ hybrid pollen donor and a *B*. *rapa* seed parent. Each of the 10 F_1_ hybrid and *B*. *rapa* were used to make BC_1_ seeds. For all of the progeny, crossability was calculated as the number of full seeds obtained per pollinated flower, and outcrossing rate was estimated by the survival (%) of seedlings after herbicide treatment. Briefly, seedlings were sprayed with 0.3% Basta (Bayer CropScience GmbH, Manheim am Rhein, Germany) at the 4–5 leaf stage and again 4 d later, and seedling survival was measured at 4–7 d after the second application.

### Confirmation of hybridization by PCR

Genomic DNA was extracted from the leaves of plants, using the CTAB method [27], and PCR analysis was performed using primer sets for *bar* (forward primer: 5′-TCTGCACCATCGTCAACCACTACAT-3′; reverse primer 5′-CTGAAGTCCAGCTGCCAGAAAC CCA-3′) or the partial 35S promoter region and *BrAGL20* (forward primer: 5-GACGCACAATCCCA CTATC-3′; reverse primer: 5′-TCACTTTCTTGAAGAACAAGG-3′). Because *BrAGL20* gene has been cloned from *B*. *rapa* and transformed to *B*. *napus*, to detect the target gene of *BrAGL20* from the F_1_ hybrid, a part of *35S* promoter of the plant expression vector and *BrAGL20* gene were used as the PCR amplification site. The PCR reactions contained 50 ng genomic DNA, 1× f-Taq buffer (Solgent, Republic of Korea), 50 μM dNTP mix, 1× Band Doctor (Solgent), 1.5 U f-Taq DNA polymerase (Solgent), and 25 pM of each primer in a total volume of 50 μl. The reaction conditions for *bar* amplification were as follows: pre-denaturation at 95°C for 4 min; 39 cycles of denaturation at 95°C for 15 s, annealing at 55°C for 30 s, and extension at 72°C for 30 s; and a final extension at 72°C for 7 min. The reaction conditions for amplification of the partial *35S* promoter and *BrAGL20* were as follows: pre-denaturation at 95°C for 4 min; 39 cycles of denaturation at 95°C for 15 s, annealing at 53°C for 30 s, and extension at 72°C for 1 min; and a final extension at 72°C for 7 min. Amplifications were performed in a PTC-100 thermal cycler (Bio-Rad, Hercules, CA, USA), and the amplified products were electrophoresed on 0.8% agarose gels, stained with EtBr, and visualized using a UV transilluminator.

### Confirmation of hybridization by flow cytometry

Fresh seedling leaves were chopped with a sharp razor blade in nuclei extraction buffer (solution A of the High Resolution Kit for Plant DNA; Partec Gmbh, Munster, Germany). After filtration through a 30-μm nylon sieve, the nuclei were stained with a solution that contained 4,6-diamidino-2-phenylindole-2HCl (i.e., DAPI; solution B of the High Resolution Kit for Plant DNA; Partec GmbH) and analyzed, using a PAS flow cytometer (Partec Gmbh). To estimate ploidy level, the fluorescent intensity peak of each sample was compared to the peaks of plants with known ploidy levels. One month later, leaves of the same plants were re-analyzed for confirmation.

### Comparing of flowering time in transgenic *B*. *napus* and F_1_ hybrid

Seeds were sown in 4-inch pots containing a vermiculite. All plants were grown in a greenhouse under controlled temperatures (22/17°C day/night). The flowering responses were measured in terms of anthesis time (number of days until the first flower in the primary inflorescence opened). All experiments were done three times using at least 10 samples each time.

### RNA extraction and real-time RT-PCR

Apical meristem samples were collected from *B*. *napus*, *B*. *rapa*, transgenic *B*. *napus*, and the F_1_ hybrid once a week for six weeks. Total RNA was prepared from the apical meristem samples using Trizol (Sigma-Aldrich, St. Louis, MO, USA), and 100 μg aliquots of the preparations were treated with RNase-Free DNase (Qiagen, Ontario, Canada). cDNA was generated by reverse transcribing 3 μg aliquots of the purified RNA in 50 μl reactions, using the ProSTAR First-Strand RT-PCR system (Stratagene, La Jolla, CA, USA) in the presence of oligo (dT) primers for 1 h at 37°C, and the reactions were terminated with heat inactivation at 70°C for 15 min.

To analyze the expression of the *BrAGL20* transgene and other floral-related genes (i.e., *AGL24*, *LFY*, and *AP1*), real-time RT-PCR was performed, using gene-specific primers: (*BrAGL20* forward: 5′-ATGGTGAGGGGCAAAACTCA-3′; *BrAGL20* reverse: 5′-TCACTTTCTTGAGAACA AG-3′; *AGL24* forward: 5′-ATGGCGAGAGAAGATAAGG-3′; *AGL24* reverse: 5′-TCATTCCCAA GATGGAAGCCC-3′; *LFY* forward: 5′-ATGGATCCTGAAGGTTTCACG-3′; *LFY* reverse 5′-TTAA ACCCCAAAGCGTCCAGA-3′; *AP1* forward: 5′-ATGGGAAGGGGTAGGGTTCAA-3′; *AP1* reverse: 5′-TCATGCGGCGAAGCAGCCAAG-3′).

All qPCR experiments were performed in a CFX real time system (Bio-Rad, USA) employing SYBR Green real-time RT-PCR Master Mix (Toyobo, Osaka, Japan). The reaction mixtures contained 5 μl diluted cDNA template (1:25), 10 μl 2× SYBR Green real-time PCR Master Mix (Toyobo), and 0.5 μM of each primer in a final volume of 20 μl. Amplification curves were generated using an initial denaturing step at 95°C for 3 min; followed by 40 cycles of 95°C for 15 s, 56°C for 15 s, and 72°C for 20 s; and melting curve analysis was performed at the end of the cycles (65 to 95°C at 0.2°C/s with continuous fluorescence readings), in order to ensure that single PCR products were obtained. To normalize the results, the *B*. *napus* actin gene (NCBI acc. no. KM881429) [28] was used as an internal standard. All real-time RT-PCR reactions were repeated in triplicate, using RNA isolated from three biological replicates, and the gene expression results were analyzed using CFX Manager Software version 1.0 (Bio-Rad).

### Structural analysis of pollen and stomata

To observe pollen grains *via* scanning electron microscopy (SEM), anthers were fixed in formalin—acetic acid—alcohol (FAA) for 48 h, dehydrated with increasing concentrations of ethanol (70%, 85%, 95%, and 100%), and fixed on a worktable with double-sided adhesive. The samples were then subjected to CO_2_ critical point drying, sprayed with gold, and imaged using a Philips XL30 ESEM (Philips Co., Netherlands) at 30 kV [29]. Ten images of each sample were randomly selected, and the size and morphological character of the pollen grains were analyzed. In order to observe the stomata, leaf samples were cut to 0.5 cm^2^, processed identically to the pollen, and observed *via* SEM.

### Lipid extraction and fatty acid methyl ester analysis

*B*. *rapa* elongates oleic acid (C18:1) to higher-level monounsaturated fatty acids (MUFAs) through the activity of *FATTY ACID ELONGASE 1 (FAE1)*, which results in a seed oil content of ~41.1% erucic acid (C22:1) and eicosenomic acid (C20:1). However, the canola-type *B*. *napus* used in the present study possesses a mutated *FAE1*, which results in the near absence of C20 or higher-level MUFAs. Therefore, the F_1_ hybrid will likely contain about half the amount of higher-level MUFAs between *B*. *rapa* and transgenic *B*. *napus*. To confirm this, we analyzed the fatty acid composition of lipids from the seeds of *B*. *napus*, *B*. *rapa*, transgenic *B*. *napus*, and F_1_ hybrids collected at 10, 20, 30, 40, and 50 DAP.

For lipid extraction, about 100 mg of developing or mature seeds from each plant were crushed in glass tubes with a steel rod, and 0.5 ml toluene and 0.5 ml 5% H_2_SO_4_ (v/v) in methanol were added to each sample. The tubes were sealed with polytetrafluoroethylene-lined screw caps, and oil extraction and transmethylation were achieved by incubating the samples at 85°C for 90 min, after which 1 ml of 0.9% NaCl (w/v) and 0.5 ml n-hexane were added and the samples were shaken vigorously. The samples were centrifuged at 4000 rpm and room temperature for 2 min. The upper phases, which consisted of fatty acid methyl esters, were transferred to gas chromatograph vials and analyzed, using a GC-2010 plus gas chromatograph (Shimadzu, Kyoto, Japan) with a 30 m × 0.25 mm (i.d.) HP-FFAP column (Agilent Technologies, Santa Clara, CA, USA), while increasing the oven temperature from 190°C to 230°C at 3.5°C/min. Nitrogen was used as the carrier gas, with a flow rate of 1.3 ml/min.

### Comparison of *FAE1* sequences

The two paralogous genes, *Bn*.*FAE1-A8* and *Bn*.*FAE1-C3*, which are involved in the synthesis of erucic acid and are genetically linked, were cloned from *B*. *napus*, *B*. *rapa*, transgenic *B*. *napus*, and the F_1_ hybrids and were evaluated for their relevance to the erucic acid content of F_1_ hybrid seeds.

Genomic DNA was extracted from *B*. *napus*, *B*. *rapa*, transgenic *B*. *napus*, and the F_1_ hybrid, using the CTAB method [27], and the *FAE1* paralogs were amplified using gene-specific PCR primers (FAE1-A8 forward: 5′-GGCACCTTTCATCGGACTAC-3′; FAE1-A8 reverse: 5′-GATAGAACTCGGGGTTTTAGTTG-3′; FAE1-C3 forward: 5′-GGCACCTTTCATCGGACTAC-3′; FAE1-C3 reverse: 5′-TTAACAGAAGATCCTTAACCCC-3′). The PCR reactions contained 50 ng genomic DNA, 4 μl 2.5 mM dNTP mix, 1 U ExTaq polymerase (TaKaRa Bio Inc., Otsu, Japan), 1× ExTaq buffer (TaKaRa Bio Inc.), and 10 pmol of each primer in a total volume of 50 μl. The amplification conditions included an initial denaturation step at 94°C for 4 min; followed by 39 cycles of denaturation at 95°C for 15 s, annealing at 55°C for 30 s, and extension at 72°C for 1.5 min; and a final extension step at 72°C for 7 min. Amplifications were performed in a PTC-100 thermal cycler (Bio-Rad).

## Results

### Outcrossing rate analysis between *Brassica rapa* and transgenic *B*. *napus*

Hand pollination of 404 *B*. *rapa* flowers resulted in the formation of 301 pods and, thus, a pod setting ratio of 74.5%, and a mean of 17 seeds were acquired from each pod (Table 1). The pod setting ratio of self-pollinated *B*. *rapa* was 59.4%, and the crossability index was 10.7. Meanwhile, for self-pollinated *B*. *napus* and transgenic *B*. *napus*, the pod setting ratios were 68.6% and 82.8%, respectively, and the crossability indices were 22.1 and 15.2. In addition, all of the herbicide resistant F1 hybrids indicated 100% outcrossing. However, out of the 2,457 F_1_ hybrid seeds, 600 were cracked (24.4%), owing to precocious germination and seed abortion. Meanwhile, 0.2% and 0% of cracked seeds were observed from self-pollination of *B*. *napus* and *B*. *rapa*, respectively.

**Table 1:** Outcrossing rate analysis of hybridization (F1) and backcross (BC1) progeny.

	Plant type	No. pollinated flowers	No. pods	Pod setting ratio (%)	Total no. seeds (no. cracked seeds)	Crossability index (seeds/pod)	Cracked seeds(%)	Outcrossing rate(%)
*Brassica rapa* L. ‘Jangkang’(selfing)	parent	256	152	59.4	1,629	10.7	0.7	-
*B*. *napus* L. ‘Youngsan’ (selfing)	parent	363	250	68.8	5,526	22.1	1.0	-
TG *B*. *napus* (selfing)	parent	337	279	82.8	4,191 (7)	15.2	0.2	-
*B*. *rapa* ‘Jangkang’*♀* × TG *B*. *napus♂*	F1	404	301	74.5	2,457 (600)	17	24.4	100
F1 hybrid (selfing)	F2	4,053	-	-	-	-	-	-
*B*. *rapa*‘Jangkang’*♀* ×F1 hybrid*♂*	BC1	2,294	492	21.5	1,956 (460)	4	23.5	50

TG *B*. *napus*, early flowering transgenic *B*. *napus* L. ‘Youngsan’

### Confirmation of hybridization by PCR and flow cytometry

To study the gene flow from transgenic *B*. *napus* to the F_1_ plant, PCR analysis was carried out for all F_1_ plants showing herbicide resistance (S1 Fig). A 445-bp band that represented the *bar* gene was detected in the PCR amplification products of all of the herbicide resistant F_1_ hybrids, as was the 738 bp band that represented *BrAGL20*. In addition, the results of flow cytometry indicated that the F_1_ hybrid (channel 150) was a triploid hybrid, since *B*. *napus* and transgenic *B*. *napus* exhibited peaks at channel 200 and *B*. *rapa* exhibited a peak at channel 100 (S2 Fig).

### Pollen morphology

**Fig 1:**
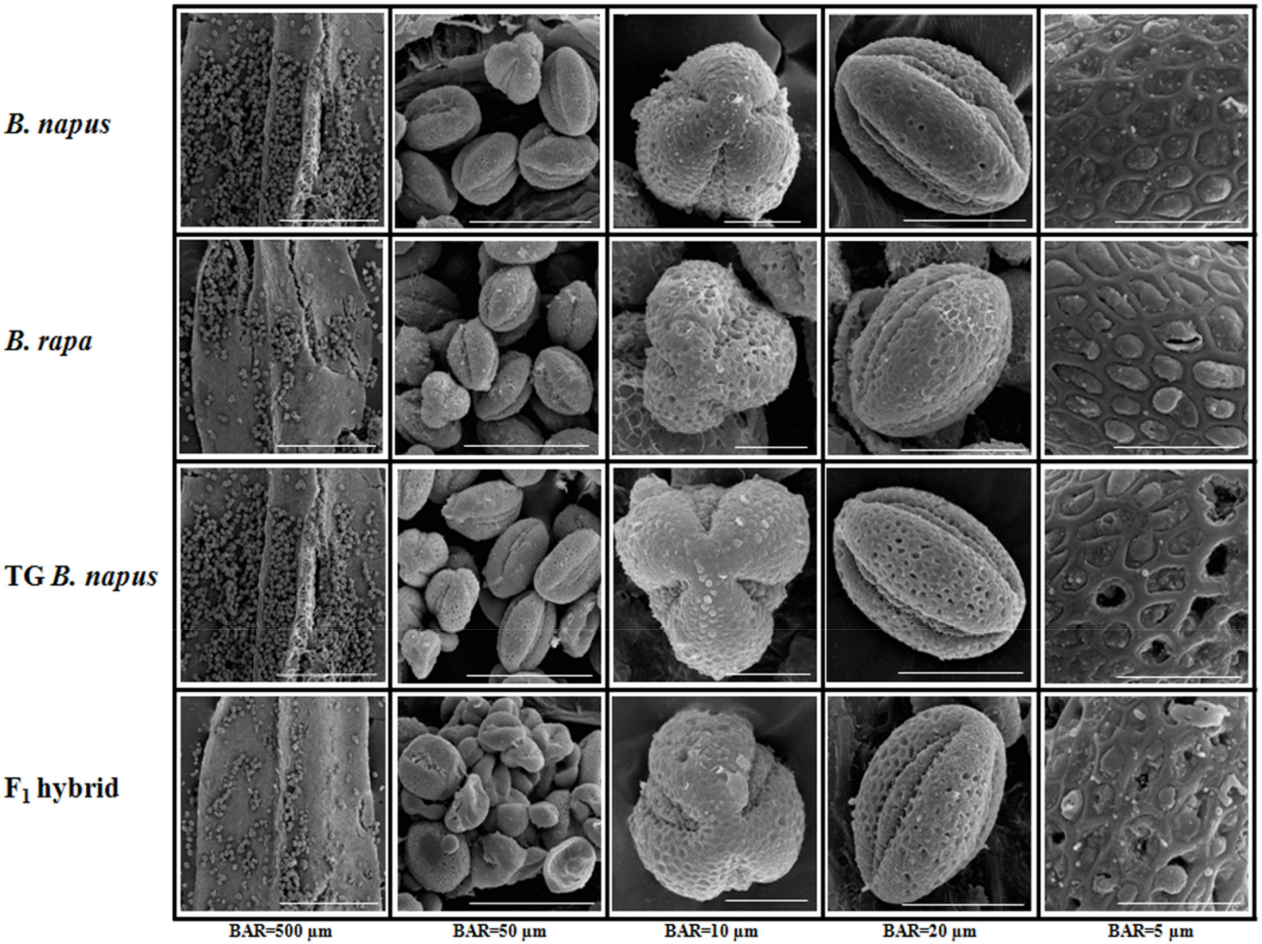
Scanning electron microscope of pollen grains from *Brassica napus*, *B*. *rapa*, transgenic (TG) *B*. *napus*, and the F_1_ hybrid. Column 1, pollen sacs; column 2, pollen grains; column 3, archopyle; column 4, enlarged image of pollen grains; column 5, pollen grain surface.

To investigate whether the hybridization between *B*. *rapa* and transgenic *B*. *napus* would have induced changes in the shape and size of pollens, the pollens of F_1_ hybrids were compared with both parents, *B*. *rapa* and transgenic *B*. *napus* using SEM (Fig 1, Table 2). The length and width of transgenic *B*. *napus* pollen were less than the pollen of *B*. *napus*, whereas the F_1_ pollen was shorter than the parental mean and ~11.8% wider. In addition, about 40% of the F_1_ pollen grains were smaller average (200 μm^2^) or exhibited abnormal morphology (Fig 1).

**Table 2:** Influence of ploidy on floral characteristics of *Brassica napus*, *B*. *rapa*, transgenic *B*. *napus*, and F_1_ hybrid.

	Ploidy level	Chromosome no.	Normal pollen		Small pollen	
			Length (μm)	Width (μm)	Length (μm)	Width (μm)
*B*. *napus* L. ‘Youngsan’	4n	38	45.1 ± 1.1^d^	19.1 ± 0.9^c^	-	-
*B*. *rapa* L. ‘Jangkang’	2n	20	35.7 ± 1.2^a^	16.1 ± 0.8^a^	-	-
TG *B*. *napus*	4n	38	40.7 ± 0.6^c^	17.7 ± 0.7^b^	-	-
*B*. *rapa♀* ×TG *B*. *napus*♂	3n	29	37.4 ± 1.7^b^	18.9 ± 1.4^c^	20.1 ± 0.7	15.8 ± 1.2

Values indicate the mean ± standard deviation of three replicates.

^a,b,c^ Alphabet which is different from each other within same column means significantly different (Duncan’s test, p *<* 0.05).

TG *B*. *napus*, transgenic *B*. *napus* L. ‘Youngsan’; *B*. *rapa♀* × TG *B*. *napus*♂, F1 hybrid of *B*. *rapa* L. ‘Jangkang’ and TG *B*. *napus* L. ‘Youngsan’. n, Number of chromosomes in gametic cells.

### Correlation between ploidy and stomatal density

**Fig 2:**
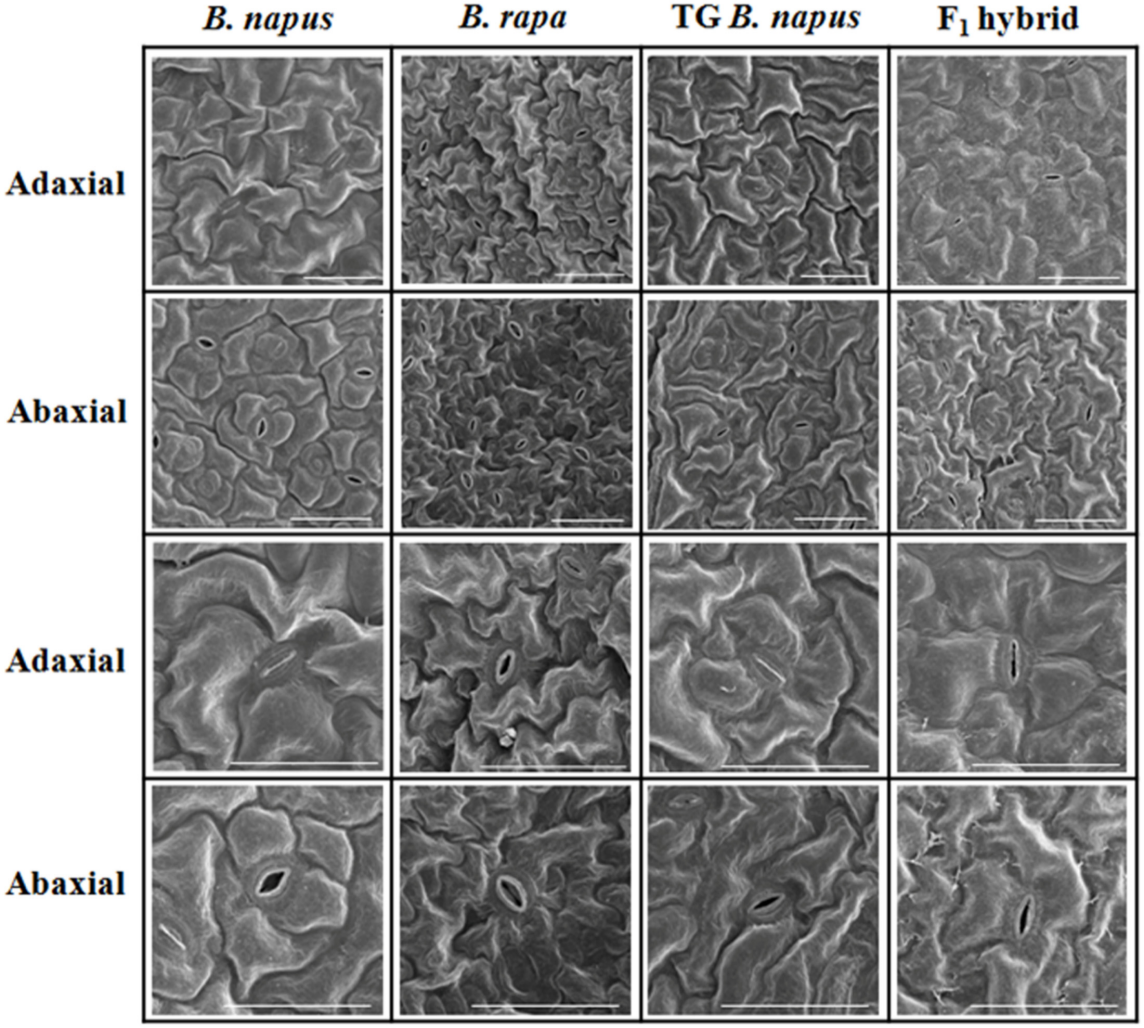
Scanning electron microscope of the adaxial and the abaxial leaf surfaces of *Brassica napus*, *B*. *rapa*, transgenic (TG) *B*. *napus*, and F_1_ hybrids. Bar indicates 50 μm. The two lower rows show enlarged images of two upper rows.

Stomatal apparatus may serve as an important phylogenetic key for plants and thus *B*. *rapa*, *B*. *napus*, transgenic *B*. *napus* and F_1_ hybrid were compared to identify any differences in the number and form of stomatal apparatus using SEM. The number of stomata on the adaxial and abaxial leaf surfaces was different in each of the plants, and the difference was greatest in *B*. *rapa* (Fig 2, Table 3).

**Table 3:** Influence of ploidy on various floral characteristics in *Brassica napus*, *B*. *rapa*, transgenic *B*. *napus* and F_1_ hybrid.

	Ploidy level	Chromosome no.	No. of stomatal apparatus	
			Adaxial	Abaxial
*B*. *napus* L. ‘Youngsan’	4n	38	*2.0±0.0^a^	2.7±0.6^a^
*B*. *rapa* L. ‘Jangkang’	2n	20	3.7±0.6^c^	5.7±0.6^c^
TG *B*. *napus*	4n	38	2.3±0.6^a^^b^	3.3±0.6^a^^b^
*B*. *rapa♀* × TG *B*. *napus*♂	3n	29	3.0±0.0^b^^c^	4.3±0.6^b^

*, Values indicate the mean number of stomatal apparatus ± standard deviation of three replicates.

^a,b,c^ Alphabet which is different from each other within same column means significantly different (Duncan’s test, p *<* 0.05).

TG *B*. *napus*, early flowering transgenic *B*. *napus* L. ‘Youngsan’; *B*. *rapa♀* × TG *B*. *napus*♂, F_1_ hybrid between *B*. *rapa* L. ‘Jangkang’ and TG *B*. *napus* L. ‘Youngsan’.

In *B*. *rapa*, the number of stomata within 100 μm^2^ surface area was 3.7 on the adaxial surface and 5.7 on the abaxial surface. In *B*. *napus*, the number of stomata within 100 μm^2^ surface area was 2 on the adaxial surface and 2.7 on the abaxial surface, and in transgenic *B*. *napus*, the number of stomata were 2.3 on the adaxial surface and 3.3 on the abaxial surface. In the F_1_ hybrid, the number of stomata was 3 on the adaxial surface and 4.3 on the abaxial surface, which was close to the mean of *B*. *rapa* and transgenic *B*. *napus*.

### Flowering time and expression of flowering-related genes in F_1_ hybrids

**Fig 3:**
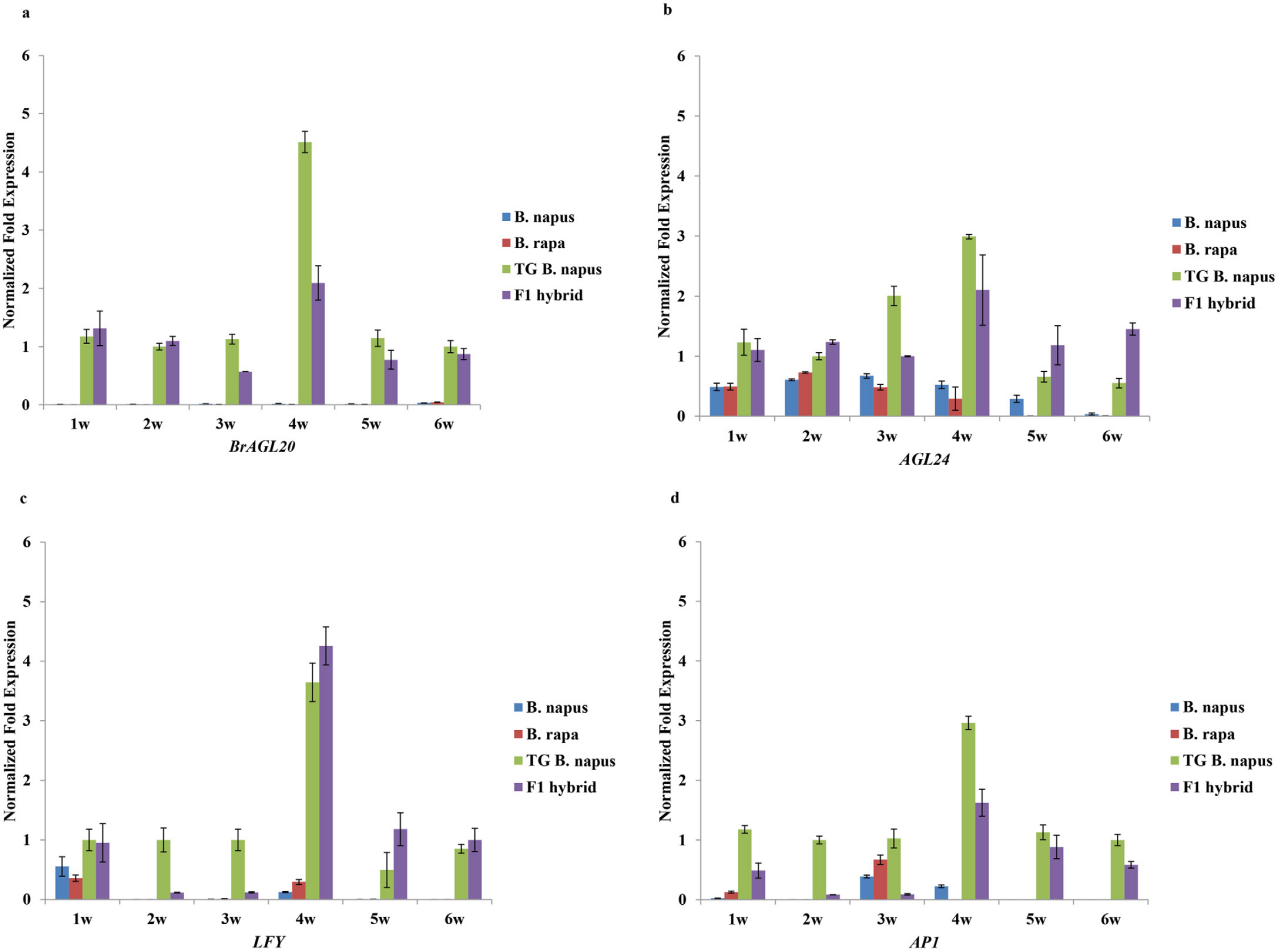
Real-time RT-PCR quantification of (A) *BrAGL20*, (B) *AGL24*, (C) *LFY*, and (D) *AP1* expression in *Brassica napus*, *B*. *rapa*, transgenic (TG) *B*. *napus*, and F_1_ hybrid apical meristems collected over six weeks. Values indicate the mean (n = 3) accumulation of each gene transcript, relative to the corresponding actin gene levels.

When *B*. *napus*, *B*. *rapa*, transgenic *B*. *napus*, and the F_1_ hybrid were planted in a greenhouse, transgenic *B*. *napus* bloomed at 33 d after planting, whereas the F_1_ hybrid bloomed at 37 d after planting. In addition, under the same conditions, the expression of *BrAGL20* in the apical meristems of transgenic *B*. *napus* and the F_1_ hybrids exhibited peaks at week 4 that were about 4.5 and 2.1 times higher than that of the actin internal control gene, after which they decreased (Fig 3). Similarly, the expression of *AGL24* in transgenic *B*. *napus* and the F_1_ hybrids increased at week 4, to 3 and 2.1 times that of actin, respectively, after which they decreased. *LFY* expression also peaked at week 4, to 3.6 and 4.3 times that of actin, in transgenic *B*. *napus* and the F_1_ hybrids, respectively, and *AP1* expression peaked to 3 and 1.6 times that of actin.

### Fatty acid composition

**Fig 4:**
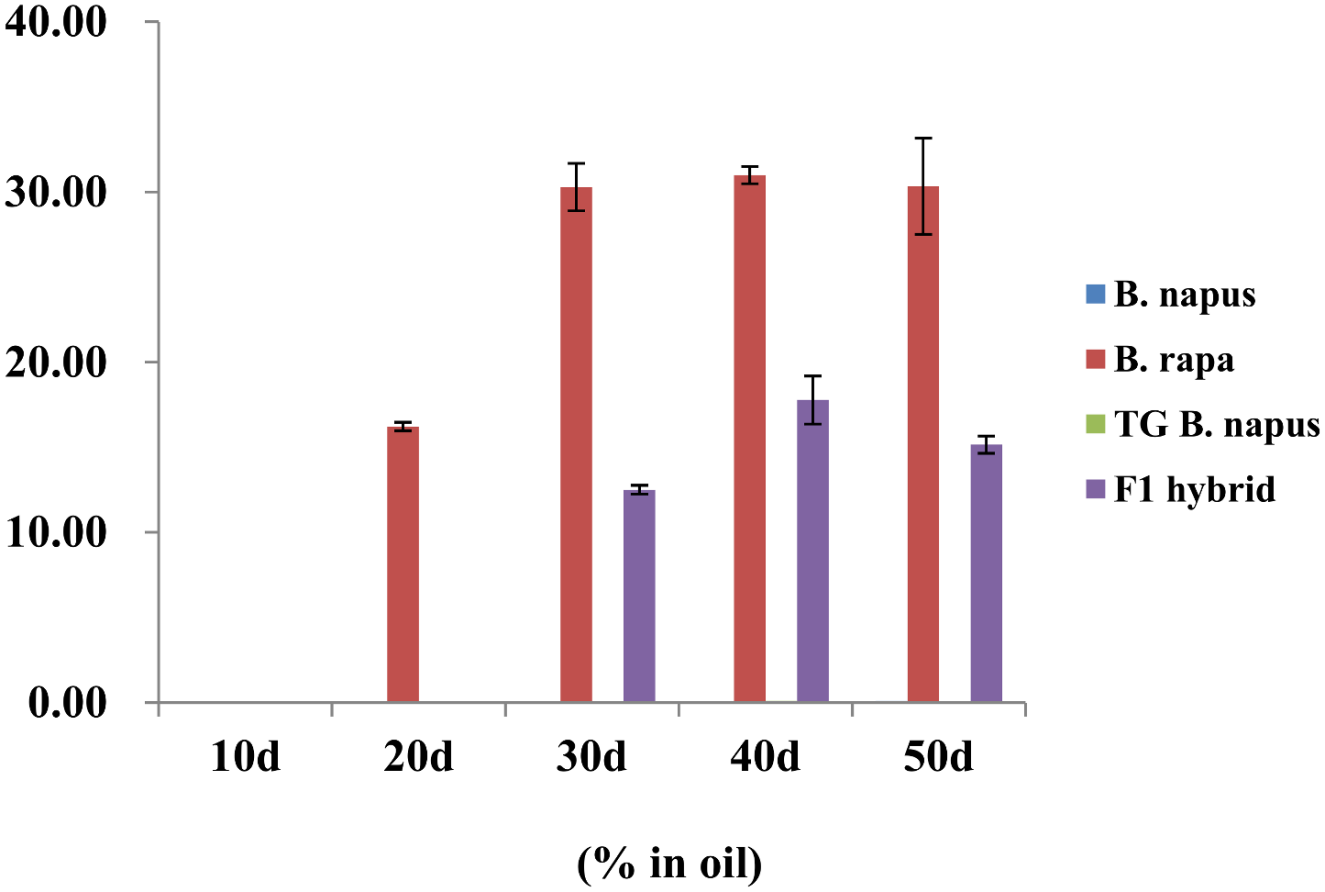
Erucic acid content (%) of *Brassica napus*, *B*. *rapa*, transgenic (TG) *B*. *napus*, and F_1_ hybrid seeds at 10, 20, 30, 40, and 50 days after pollination.

The fatty acid composition of *B*. *napus* seeds was similar to that of transgenic *B*. *napus* seeds (Fig 4, S1 Table), and at 50 DAP, the fatty acid composition of *B*. *napus* and transgenic *B*. *napus* seeds was 6.1% and 5.3% palmitic acid (C16:0), respectively, 3.1% and 2.8% stearic acid (C18:0), 60.7% and 69.4% oleic acid (C18:1), respectively, 19.2% and 14.1% linoleic acid (C18:2), 6.7% and 4.5% linolenic acid (C18:3), 1.5% and 1.4% eicosenoic acid (C20:1), and 0.09% and 0.06% erucic acid (C22:1). At 50 DAP, the fatty acid composition of *B*. *rapa* seeds was 3.3% C16:1, 1.3% C18:0, 33.3% C18:1, 12.3% C18:2, 5.4% C18:3, 10.8% C20:1, and 30.3% C22:1. At 50 DAP, the fatty acid composition of F_1_ hybrid seeds was 4.6% C16:0, 1.5% C18:0, 35.9% C18:1, 19.4% C18:2, 6.6% C18:3, 14.4% C20:1, and 17.8% C22:1.

### Characterization of *Bn*.*FAE1-A8* and *Bn*.*FAE1-C3*

**Fig 5:**
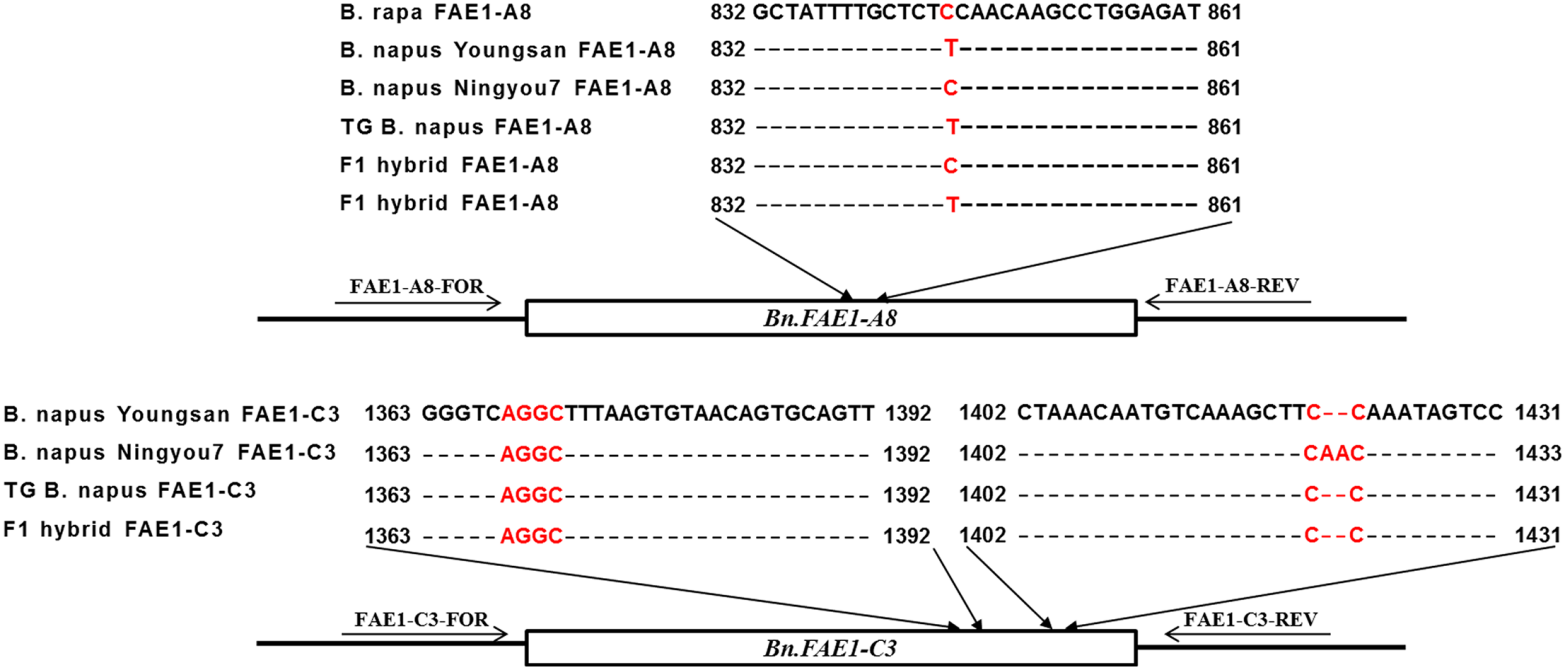
Nucleotide sequence comparison of *BnA8*.*FAE1* and *BnC3*.*FAE1* from *Brassica napus*, *B*. *rapa*, transgenic (TG) *B*. *napus*, and F_1_ hybrids.

*Bn*.*FAE1-A8* from *B*. *napus* and transgenic *B*. *napus* exhibited a C-to-T substitution at 845 bp, as well as a deletion of two adenine residues at 1,422–1,423 bp (Fig 5). However, no changes were observed in the *B*. *rapa* sequences, and the F_1_ hybrids exhibited a ~50:50 ratio of C and T at 845 bp.

### Seed production of the BC1 generation

The hand pollination of 2,294 *B*. *rapa* flowers with F_1_ hybrid pollen resulted in the formation of 492 pods (21.5% pod setting rate), with an average of four seeds per pod. In the bioassay of BC_1_ progeny, 50% of BC_1_ progeny exhibited resistance to 0.3% Basta (outcrossing rate; Table 1), and similar to the F_1_ hybrid seeds, 460 of 1,956 seeds (23.5%) were cracked. In addition, both the 445 bp band that indicated the amplification of *bar* and the 738 bp band that indicated the amplification of the partial *35S* promoter and *BrAGL20* were detected in the PCR amplification products of all of the herbicide-resistant BC_1_ progeny (S1 Fig).

## Discussion

### Outcrossing rate between *B*. *rapa* and transgenic *B*. *napus*

Although a number of studies have investigated the gene flow of transgenic *B*. *napus* to other species in the *Brassicaceae*, the controversy over the environmental impact of GMOs remains. The facts that the wild relative species of *Brassicaceae* easily hybridized with transgenic *B*. *napus* are greater in number compared to other transgenic crops, and they are largely distributed to the surrounding environment including nearby roadsides to cause environmental issues are the cause of such controversy [14, 1, 19]. However, the acreage of transgenic *B*. *napus* increases each year, owing to its economic benefits, and the increase is expected to continue.

The hand pollination of a number of *B*. *rapa* cultivars with transgenic *B*. *napus* pollen resulted in a wide range of crossability, depending on the *B*. *rapa* cultivar [12]. In the present study, 17 seeds were harvested per pod, which indicated moderate crossability between the maternal and paternal parents. Variability in the crossability of different *B*. *rapa* genotypes is thought to be influenced by the attachment of pollen grains to the stigma, pollen tube penetration, and the abortion of developing embryos [12]. In addition, another study reported that the amount of callose accumulated by papilla cells during pollination between *B*. *rapa* and *B*. *napus* forms a reproductive barrier that affects crossability [30].

In the present study, the proportion of seeds that were cracked by precocious germination and abortion was 24.5%, which is lower than that observed by Xiao et al. [12]. Cracked seeds can be attributed to three main causes. When seeds were observed at 10–50 DAP, precocious germination was observed between 10 to 20 DAP when *B*. *rapa* was used as the maternal parent and transgenic *B*. *napus* was used as the pollen donor (S3 Fig). The phenomenon of precocious germination in hybrids of *B*. *rapa* and *B*. *napus* been reported previously [12,31,32] and is thought to be caused by seed-specific immunomodulation that switches seed maturation to seed germination [33]. The absence of precocious germination in the hybridization of *B*. *rapa* with pollen from resynthesized *B*. *napus* suggests that the phenomenon was influenced by the paternal parent [12]. The culling of seeds without normal maturation, owing to the formation of callus tissues during seed development, was another cause of cracking, and although the cause of the phenomenon is still unclear, hormonal imbalance are likely responsible. Lastly, seed abortion, a phenomenon of stalled seed development after fertilization, was considered to be cracked. The formation of cracked seeds was also observed in the backcross of the F_1_ hybrid to *B*. *rapa*, with a cracked seed ratio (23.5%) that was similar to that of the F_1_ hybrids. The size of F_1_ hybrid seeds was relatively small compared to that of parent seeds, and the thousand-seed weight was lighter than that of the parents, as well (S2 Table). Small seed size may be a disadvantage for fitness, since it could negatively affect seed emergence, initial seedling size, and initial competitiveness over other plants [34]. The fitness of small seed-derived plants might also be reduced under highly dense or shaded conditions, or in response to drought or herbivory. However, despite the lower thousand-seed weight and seed size of the F_1_ hybrids in the present study, 100% germination was observed in un-cracked seeds, and the plants also exhibited great vitality.

### Correlation between ploidy level and stomatal density

Stomatal density, guard cell length, and stomatal plastid number are often used as morphological markers for identifying the ploidy level of plants [35–38]. In the present study, the adaxial and abaxial leaf surfaces of *B*. *napus*, *B*. *rapa*, transgenic *B*. *napus*, and F_1_ hybrids in the same development phase were examined *via* SEM. The stomata of the adaxial and abaxial leaf surfaces were different in all four plants, and those of *B*. *napus* and transgenic *B*. *napus* were also different. The stomata of the F_1_ hybrid exhibited an intermediate form of *B*. *rapa* and transgenic *B*. *napus*. In general, as ploidy increases, the density of guard cells and epidermal cells are reported to decrease, whereas the length of the guard cells is reported to increase [37,38]. In the observation of guard cells for diploid and triploid *Citrus clementine*, there was a positive correlation with ploidy level and stomatal cell length and width and a negative correlation with ploidy level and stomatal density [39]. As the result of analyzing the number of stomata within 100 μm^2^ leaf surface area, the present study found that the number of stomata decreased as ploidy level increased, and the F_1_ hybrids exhibited intermediate values, when compared to the parent, which is consistent with the data reported by Padoan et al. [39]. The increased guard cell density, which is related to the transpiration rate of guard cells, is required for the movement of water and nutrients [40], and leaf water loss is higher in diploid plants than in triploid plants [39]. Therefore, it remains to be studied that the relationship between the fitness of F_1_ hybrids and the transpiration rate of their guard cells be analyzed under various weather conditions.

### Expression of flowering-related genes in F_1_ hybrids

Transgenic *B*. *napus* started flowering on day 33 on average, whereas the F_1_ hybrids started flowering on day 37. Both the F_1_ hybrids and the transgenic *B*. *napus* bloomed in the greenhouse without vernalization. The difference in flowering time between transgenic *B*. *napus* and the F_1_ hybrids was considered to result from the expression levels of *BrAGL20*. Transgenic *B*. *napus*, which was homozygous for *BrAGL20*, exhibited a level of *BrAGL20* expression that was ~2.1 times that of the F_1_ hybrids, which were hemizygous for *BrAGL20*. This additive transgene expression was also observed in F_1_ hybrids of wild *B*. *rapa* and transgenic *B*. *napus* that was homozygous for GFP [41]. The expression level of *AGL20* in *Arabidopsis* is known to play an important role in regulating flowering time, and the gene functions as the key floral activator that integrates several floral inductive pathways [25]. In transgenic *B*. *napus*, flowering time is increasingly advanced as the expression level of *BrAGL2*0 increased [26].

Flowering time genes that regulate floral transition have been reported to regulate the activity of floral meristem identity genes, including *LFY* and *AP1*(*APETALA1*) [42–44]. *SOC1* regulates *LFY* expression by directly binding to the *LFY* promoter, and the LFY protein activates *AP1* expression by binding to the *cis*-element of *AP1*. However, Lee et al. [45] suggested that heterodimerization of *SOC1* and *AGL24* is the key mechanism of *LFY* expression, since *LFY* expression appears in tissues that express *SOC1* and *AGL24* simultaneously.

In the present study, the expression of *AGL24*, *LFY*, and *AP1* was found to increase, along with *BrAGL20*, in the F_1_ hybrid and transgenic *B*. *napus* from week 4, compared to *B*. *napus* and *B*. *rapa*. Therefore, the regulation of *AGL24*, *LFY*, and *AP1* by *BrAGL20* expression was likely involved in the early flowering of transgenic *B*. *napus* and the F_1_ hybrids, and the flowering pattern of these plants is thought to have resulted from the additive expression of *BrAGL20*. The expression peaks of flowering-related genes decreased in the samples from week 5 and week 6.

### Fatty acid composition and *FAE1* characterization in F_1_ hybrids

The fatty acid composition of *Brassicaceae* is more genetically diverse than that of other major vegetable oil crops, which suggests that the fatty acid composition can be used as a taxonomic character [46,47]. Among the fatty acids, erucic acid is known to create cardiotoxicity [48]. The *B*. *napus* that was developed for edible use has low content of erucic acid, but the *B*. *rapa* has erucic acid. Therefore, we aimed to investigate how much content of erucic acid is accumulated in F_1_ hybrid seeds, assuming when F_1_ hybrid seeds were released to the environment. In the present study, the fatty acid composition of *B*. *napus*, *B*. *rapa*, transgenic *B*. *napus*, and F1 hybrid seeds was analyzed to determine changes in the fatty acid composition of the F_1_ hybrid seeds. Erucic acid exhibited the greatest change, and the content of erucic acid in the F_1_ hybrid seeds was intermediate between *B*. *rapa* and transgenic *B*. *napus*.

The modern oilseed *Brassica napus*, which is also called canola, was discovered along with the germplasm of Liho, which is a German spring forage cultivar with low content of erucic acid that has been used as healthy edible oil [49, 50]. The erucic acid synthesis in *B*. *napus* seeds is mainly controlled by two genes with additive effect [51], which are the two linked paralogs *Bn*.*FAE1-A8* and *Bn*.*FAE1-C3* [52–55]. As reported by Wang et al. [56], the cysteine residue at 845 bp in *BnFAE1-A8* is essential for gene function, and its substitution results in loss of function. In addition, the erucic acid content was decreased when by deletions at two sites in *BnFAE1-C3*: the AA site at 1,422–1,423 bp and the AGGC site at 1368–1371 bp. In the analysis of *B*. *napus* varieties, the function of the AA site was found to be more important than the AGGC site [56].

In the present study, *BnFAE1-A8* and *BnFAE1-C3* were cloned from *B*. *rapa*, *B*. *napus*, transgenic *B*. *napus*, and F_1_ hybrids, in order to analyze their nucleotide sequences. As a result, we found that cysteine was present at the 845 bp site of *BnFAE1-A8* in *B*. *rapa* and erucic acid was synthesized at 20 DAP. However, the cysteine residue at the 845 bp site was substituted by a thiamine residue in both *B*. *napus* and transgenic *B*. *napus*, and the AA site of *BnFAE1-C3* was deleted, as well. Although the AGGC site of *BnFAE1-C3* remained, the AA site was apparently more important in the synthesis of erucic acid, as mentioned in the literature [56]. Therefore, no erucic acid was synthesized in transgenic *B*. *napus* at 10–50 DAP. Since the *BnFAE1-A8* gene in the F_1_ hybrids was inherited from both *B*. *rapa* and transgenic *B*. *napus*, the probability of either C or T at the 845 bp site was estimated as 50% each, and sequencing of a number of *BnFAE1-A8* from F_1_ hybrid, confirmed that the probability of the two variations were each 50%.

In addition, the F_1_ hybrids were verified to contain almost half the level of erucic acid in *B*. *rapa* at 30 DAP, which is when erucic acid synthesis began. Thus, the erucic acid level could be used to identify F_1_ hybrid between *B*. *rapa* and transgenic *B*. *napus*. In *B*. *rapa*, erucic acid was first detected at 20 DAP and increased by over 30% at 30 DAP. In the F_1_ hybrids, erucic acid was first detected at 20 DAP, gradually increased by about 12.5% at 30 DAP, and then increased to 50% of the total fatty acids after 30 DAP. As for *B*. *napus* and transgenic *B*. *napus*, erucic acid was not detected in any seeds at 10–50 DAP.

In the present study, the *BrAGL20* transgene was transferred to F_1_ hybrids *via* hand pollination and was expressed stably, as indicated by early flowering. Even though F_2_ progeny could not be produced *via* self-pollination of F_1_ hybrids, BC_1_ progeny was obtained by backcrossing the F_1_ hybrid to *B*. *rapa*, which confirms the potential for gene flow in nature if *B*. *rapa* is distributed in the vicinity of unintentionally generated co-flowering F_1_ hybrids. The frequency distribution of chromosome numbers during the meiosis MII stage of sesquidiploid-like F_1_ hybrids was binomially distributed [57]. Gametes with other chromosomes numbers (from n = 10 to n = 19) failed to exhibit any significant difference in competitive ability before fertilization, but the survival rate after fertilization was reported to differ greatly. BC_1_ progeny is predicted to have varying numbers of chromosomes and, as a result, to exhibit variation in the expression of the target transgene, as well as in its related phenotypic trait. It is also necessary to backcross each BC_1_ progeny to generate subsequent progenies and to investigate their environmental sustainability. In addition, the available genetic resources for *Brassicaceae* are very diverse, and thus, the characteristics of hybrids from GM canola and other *Brassicaceae* should be investigated, as well.

## Supporting Information

S1 Fig PCR analysis of *bar* and target gene in *Brassica napus*, *B*. *rapa*, transgenic *B*. *napus*, F_1_ hybrid and BC_1_ progeny. PC, positive control (DNA of plant expression vector used for transformation); NC, negative control (instead of template DNA DDW was used for PCR); YS, *B*. *napus* L. ‘Youngsan’; JK, *B*. *rapa* L. ‘Jangkang’; TG, transgenic *B*. *napus* L. cv. Youngsan; F_1_, F_1_ hybrid of *B*. *rapa* L. ‘Jangkang’ and TG *B*. *napus* L. ‘Youngsan’; BC_1_, First backcross generation of *B*. *rapa ♀* and the F_1_ hybrid ♂.
(DOCX)

S2 Fig Flow cytometry histograms, showing the ploidy levels of *Brassica napus*, *B*. *rapa*, transgenic (TG) *B*. *napus*, and F_1_ hybrids. Red arrows indicate the fluorescent intensity peaks used to determine ploidy levels.
(DOCX)

S3 Fig Seed development of *Brassica napus*, *B*. *rapa*, transgenic (TG) *B*. *napus*, and the F_1_ hybrid at 10, 20, 30, 40, and 50 days after pollination (DAP). The F_1_ hybrid seeds exhibited precocious germination at 20 days after pollination (DAP) and callus tissues at 40 and 50 DAP.
(DOCX)

S1 Table Fatty acid composition of *Brassica napus*, *B*. *rapa*, transgenic (TG) *B*. *napus*, and F_1_ hybrid seeds. Values indicate the mean fatty acid content (%) ± standard deviation of three replicates. DAP, days after pollination; B. rapa, *B*. *rapa* L. ‘Jangkang’; *B*. *napus*, *B*. *napus* L. ‘Youngsan’; TG *B*. *napus*, transgenic *B*. *napus* L. ‘Youngsan’; *B*. *rapa* × TG *B*. *napus*, F_1_ hybrid between *B*. *rapa* L. ‘Jangkang’ and TG *B*. *napus* L. ‘Youngsan’.
(DOCX)

S2 Table Seed characteristics of F1 hybrid between *B*. *rapa* and TG *B*. *napus*. Values indicate the mean ±standard deviation from three replications. TG *B*. *napus*, transgenic *B*. *napus* L. cv. Youngsan; *B*. *rapa ♀* x TG *B*. *napus*♂, F_1_ hybrid between *B*. *rapa* L. cv. Jangkang and TG *B*. *napus* L. cv. Youngsan.
(DOCX)
