# Supplementary figures and images for: Characteristics Analysis of F1 Hybrids between Genetically Modified Brassica napus and B. rapa
Source: PLoS One. 2016 Sep 15;11(9):e0162103. doi: 10.1371/journal.pone.0162103 (PMC5025156; doi:10.1371/journal.pone.0162103)

**S1 Fig.**


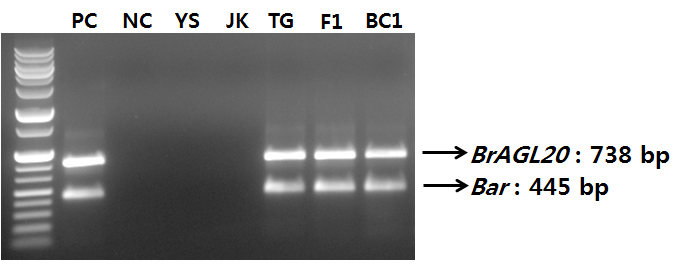

Supplement: S1 Fig — PC, positive control (DNA of plant expression vector used for transformation); NC, negative control (instead of template DNA DDW was used for PCR); YS, B. napus L. ‘Youngsan’; JK, B. rapa L. ‘Jangkang’; TG, transgenic B. napus L. cv. Youngsan; F1, F1 hybrid of B. rapa L. ‘Jangkang’ and TG B. napus L. ‘Youngsan’; BC1, First backcross generation of B. rapa ♀ and the F1 hybrid ♂. (DOCX) [file pone.0162103.s001.docx]

**S2 Fig.**


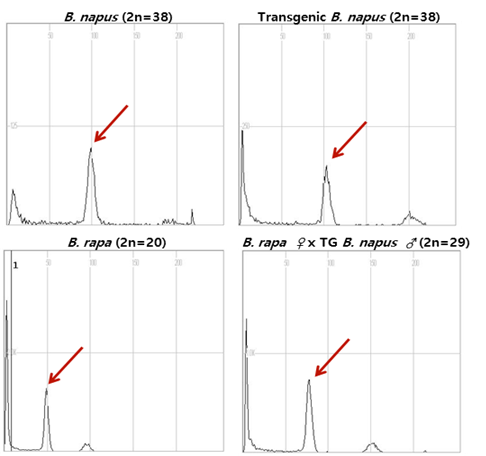

Supplement: S2 Fig — Red arrows indicate the fluorescent intensity peaks used to determine ploidy levels. (DOCX) [file pone.0162103.s002.docx]

**S3 Fig.**


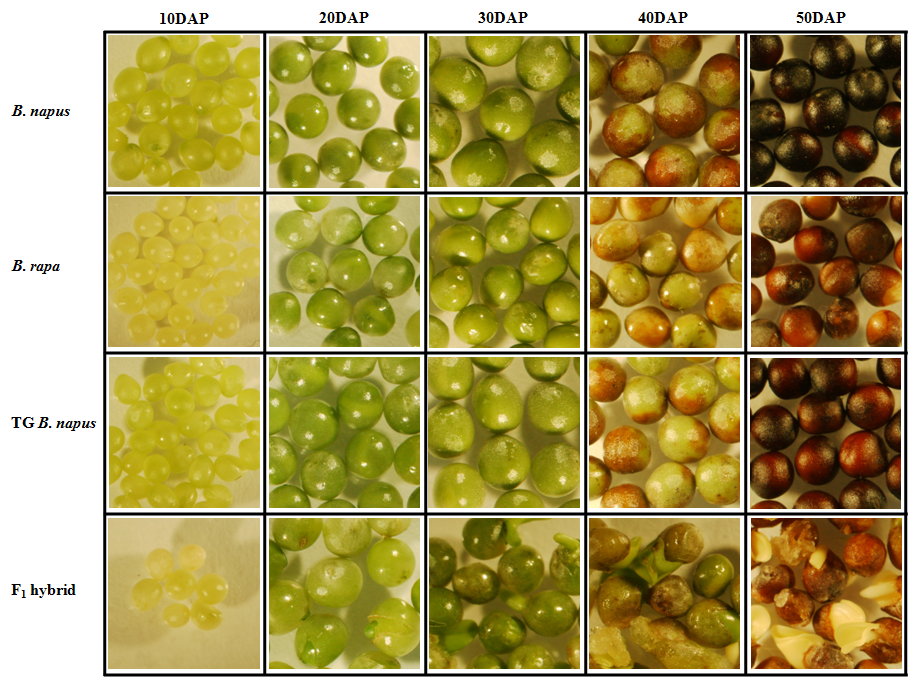

Supplement: S3 Fig — The F1 hybrid seeds exhibited precocious germination at 20 days after pollination (DAP) and callus tissues at 40 and 50 DAP. (DOCX) [file pone.0162103.s003.docx]
