# Supplementary material for: Characteristics Analysis of F1 Hybrids between Genetically Modified Brassica napus and B. rapa
Source: PLoS One. 2016 Sep 15;11(9):e0162103. doi: 10.1371/journal.pone.0162103 (PMC5025156; doi:10.1371/journal.pone.0162103)
Supplement: S1 Table — Values indicate the mean fatty acid content (%) ± standard deviation of three replicates. DAP, days after pollination; B. rapa, B. rapa L. ‘Jangkang’; B. napus, B. napus L. ‘Youngsan’; TG B. napus, transgenic B. napus L. ‘Youngsan’; B. rapa × TG B. napus, F1 hybrid between B. rapa L. ‘Jangkang’ and TG B. napus L. ‘Youngsan’. (DOCX) [file pone.0162103.s004.docx]

**S1 Table.** Fatty acid composition of *Brassica napus*, *B. rapa*, transgenic (TG) *B. napus,* and F1 hybrid seeds.

|  |  | **Fatty acid profile** | | | | | | | | | | | |
| --- | --- | --- | --- | --- | --- | --- | --- | --- | --- | --- | --- | --- | --- |
|  |  | **16:0** | **16:01** | **16:03** | **18:00** | **18:01** | **18:02** | **18:03** | **20:00** | **20:01** | **20:02** | **22:00** | **22:01** |
| **10**  **DAP** | ***B. rapa*** | **19.22±0.3** | **0.00±0.0** | **0.65±0.6** | **7.41±0.3** | **5.13±0.3** | **53.58±0.6** | **13.71±0.1** | **0.30±0.5** | **0.00±0.0** | **0.00±0.0** | **0.00±0.0** | **0.00±0.0** |
|  | ***B. napus*** | **14.1±0.2** | **1.56±0.3** | **0.80±0.1** | **4.35±0.2** | **29.41±1.3** | **37.39±0.9** | **11.95±0.7** | **0.53±0.5** | **0.00±0.0** | **0.00±0.0** | **0.00±0.0** | **0.00±0.0** |
|  | **TG *B. napus*** | **18.13±0.3** | **0.00±0.0** | **0.68±0.6** | **8.63±0.2** | **3.73±1.2** | **53.53±1.3** | **14.68±0.2** | **0.63±0.6** | **0.00±0.0** | **0.00±0.0** | **0.00±0.0** | **0.00±0.0** |
|  | ***B. rapa ×* TG *B. napus*** | **20.84±0.8** | **0.00±0.0** | **0.99±0.1** | **6.91±0.2** | **4.19±0.9** | **51.46±0.4** | **15.18±0.9** | **0.43±0.8** | **0.00±0.0** | **0.00±0.0** | **0.00±0.0** | **0.00±0.0** |
| **20**  **DAP** | ***B. rapa*** | **6.85±0.3** | **0.43±0.0** | **0.60±0.0** | **2.52±0.2** | **26.90±2.0** | **24.23±1.9** | **10.76±0.3** | **1.19±0.0** | **9.21±0.6** | **0.46±0.0** | **0.61±0.1** | **16.22±0.3** |
|  | ***B. napus*** | **8.74±0.3** | **1.04±0.0** | **0.68±0.0** | **2.60±0.2** | **47.65±2.0** | **26.24±1.9** | **11.30±0.3** | **0.77±0.0** | **0.66±0.6** | **0.02±0.0** | **0.30±0.1** | **0.00±0.0** |
|  | **TG *B. napus*** | **8.64±0.2** | **1.12±0.2** | **0.57±0.0** | **3.06±0.2** | **52.65±0.7** | **24.78±0.5** | **7.30±0.1** | **0.89±0.1** | **0.59±0.1** | **0.06±0.1** | **0.34±0.0** | **0.00±0.0** |
|  | ***B. rapa* × TG *B. napus*** | **17.39±0.9** | **1.19±0.3** | **1.42±0.1** | **4.17±0.3** | **16.22±3.0** | **42.49±1.6** | **16.44±0.6** | **0.31±0.3** | **0.36±0.3** | **0.00±0.0** | **0.00±0.0** | **0.00±0.0** |
| **30**  **DAP** | ***B. rapa*** | **4.12±0.2** | **0.28±0.0** | **0.50±0.1** | **1.36±0.0** | **22.45±0.6** | **19.36±0.4** | **10.34±0.2** | **0.79±0.1** | **9.15±0.3** | **0.54±0.0** | **0.82±0.1** | **30.28±1.4** |
|  | ***B. napus*** | **6.04±0.1** | **0.55±0.1** | **0.48±0.0** | **2.06±0.1** | **56.19±0.6** | **22.30±0.5** | **10.24±0.1** | **0.62±0.0** | **1.10±0.1** | **0.08±0.0** | **0.34±0.0** | **0.00±0.0** |
|  | **TG *B. napus*** | **6.18±0.0** | **0.61±0.0** | **0.38±0.0** | **1.94±0.1** | **62.88±0.6** | **19.81±0.7** | **6.05±0.1** | **0.66±0.0** | **1.05±0.1** | **0.07±0.0** | **0.39±0.0** | **0.00±0.0** |
|  | ***B. rapa ×* TG *B. napus*** | **6.35±0.2** | **0.95±0.1** | **0.57±0.0** | **1.89±0.2** | **30.17±0.9** | **23.63±1.0** | **10.27±0.5** | **0.81±0.1** | **11.76±0.7** | **0.59±0.0** | **0.50±0.1** | **12.51±0.3** |
| **40**  **DAP** | ***B. rapa*** | **3.49±0.3** | **0.28±0.0** | **0.38±0.0** | **1.42±0.1** | **30.76±0.4** | **14.03±0.2** | **5.97±0.1** | **0.99±0.1** | **10.09±0.2** | **0.36±0.0** | **1.25±0.0** | **30.98±0.5** |
|  | ***B. napus*** | **5.22±0.1** | **0.43±0.0** | **0.30±0.0** | **2.00±0.1** | **64.55±0.4** | **18.69±0.6** | **6.32±0.0** | **0.64±0.0** | **1.31±0.2** | **0.07±0.0** | **0.44±0.0** | **0.04±0.0** |
|  | **TG *B. napus*** | **5.06±0.2** | **0.41±0.0** | **0.31±0.0** | **3.08±0.1** | **69.15±0.8** | **13.90±0.2** | **4.55±0.2** | **1.09±0.1** | **1.57±0.2** | **0.07±0.0** | **0.69±0.1** | **0.11±0.0** |
|  | ***B. rapa ×* TG *B. napus*** | **4.90±0.3** | **0.60±0.2** | **0.31±0.0** | **1.37±0.1** | **28.31±1.9** | **22.26±0.6** | **9.35±1.4** | **0.74±0.2** | **13.22±0.6** | **0.68±0.1** | **0.48±0.2** | **17.78±1.4** |
| **50**  **DAP** | ***B. rapa*** | **3.33±0.3** | **0.29±0.0** | **0.33±0.1** | **1.36±0.1** | **33.33±2.8** | **12.31±1.0** | **5.44±0.3** | **1.11±0.3** | **10.78±0.1** | **0.27±0.0** | **1.11±0.3** | **30.33±2.8** |
|  | ***B. napus*** | **6.07±0.0** | **0.54±0.0** | **0.48±0.0** | **3.12±0.0** | **60.65±1.2** | **19.17±0.0** | **6.70±0.2** | **0.87±0.1** | **1.46±0.5** | **0.12±0.1** | **0.74±0.6** | **0.09±0.0** |
|  | **TG *B. napus*** | **5.25±0.2** | **0.48±0.0** | **0.37±0.0** | **2.76±0.1** | **69.44±0.6** | **14.01±0.2** | **4.52±0.1** | **0.93±0.1** | **1.38±0.0** | **0.05±0.0** | **0.74±0.2** | **0.06±0.0** |
|  | ***B. rapa ×* TG *B. napus*** | **4.57±0.0** | **0.43±0.0** | **0.33±0.0** | **1.53±0.0** | **35.92±0.9** | **19.36±0.1** | **6.60±0.3** | **0.78±0.1** | **14.43±0.0** | **0.48±0.0** | **0.40±0.0** | **15.16±0.5** |
| **60**  **DAP** | ***B. rapa*** | **2.84±0.1** | **0.00±0.0** | **0.00±0.0** | **1.08±0.1** | **30.07±1.1** | **11.60±0.3** | **5.54±0.2** | **0.78±0.0** | **11.37±1.0** | **0.51±0.1** | **0.88±0.2** | **35.02±0.4** |
|  | ***B. napus*** | **4.55±0.1** | **0.00±0.0** | **0.00±0.0** | **2.37±0.3** | **68.56±0.9** | **17.87±0.9** | **4.15±0.2** | **0.72±0.1** | **1.03±0.0** | **0.09±0.0** | **0.12±0.1** | **0.54±0.1** |
|  | **TG *B. napus*** | **4.22±0.0** | **0.00±0.0** | **0.00±0.0** | **1.94±0.0** | **67.17±0.4** | **16.28±0.5** | **6.42±0.1** | **0.73±0.0** | **1.45±0.1** | **0.37±0.0** | **0.86±0.1** | **0.49±0.2** |
|  | ***B. rapa ×* TG *B. napus*** | **3.92±0.1** | **0.00±0.0** | **0.00±0.0** | **1.55±0.1** | **36.96±1.1** | **14.92±0.3** | **5.76±0.3** | **0.85±0.1** | **16.58±0.1** | **0.62±0.1** | **0.83±0.2** | **17.78±0.5** |

Values indicate the mean fatty acid content (%) ± standard deviation of three replicates.

DAP, days after pollination; B. rapa, *B. rapa* L. ‘Jangkang’; *B. napus*, *B. napus* L. ‘Youngsan’; TG *B. napus*, transgenic *B. napus* L. ‘Youngsan’; *B. rapa* × TG *B. napus*, F1 hybrid between *B. rapa* L. ‘Jangkang’ and TG *B. napus* L. ‘Youngsan’.
