# Supplementary material for: Characteristics Analysis of F1 Hybrids between Genetically Modified Brassica napus and B. rapa
Source: PLoS One. 2016 Sep 15;11(9):e0162103. doi: 10.1371/journal.pone.0162103 (PMC5025156; doi:10.1371/journal.pone.0162103)
Supplement: S2 Table — Values indicate the mean ±standard deviation from three replications. TG B. napus, transgenic B. napus L. cv. Youngsan; B. rapa ♀ x TG B. napus♂, F1 hybrid between B. rapa L. cv. Jangkang and TG B. napus L. cv. Youngsan. (DOCX) [file pone.0162103.s005.docx]

S2 Table. Seed characteristics of F1 hybrid between *B. rapa* and transgenic (TG) *B. napus*

|  | Pod  length | Seed size  (mm) | Thousand seed weight (g) |
| --- | --- | --- | --- |
| *B. napus* L. ‘Youngsan’ | 7.1±0.2 | 2.4±0.2 | 4.5±0.1 |
| *B. rapa* L. ‘Jangkang’ | 5.0±0.1 | 2.3±0.2 | 4.0±0.0 |
| TG *B. napus* | 6.8±0.4 | 2.3±0.2 | 4.0±0.1 |
| *B. rapa* L. ‘Jangkang’*♀* × TG *B. napus*♂ | 4.7±0.1 | 2.2±0.1 | 3.7±0.1 |

Values indicate the mean ±standard deviation from three replications. TG *B. napus*, transgenic *B. napus* L. cv. ‘Youngsan’; *B. rapa* *♀* x TG *B. napus*♂, F1 hybrid between *B. rapa* L. cv. ‘Jangkang’ and TG *B. napus* L. cv. ‘Youngsan’
